# Differential Contribution to Neuroendocrine Tumorigenesis of Parallel Egfr Signaling in Cancer Cells and Pericytes

**DOI:** 10.1177/1947601909358722

**Published:** 2010-02

**Authors:** Olivier Nolan-Stevaux, Morgan C. Truitt, Jessica C. Pahler, Peter Olson, Cristina Guinto, David C. Lee, Douglas Hanahan

**Affiliations:** 1 Helen Diller Family Comprehensive Cancer Center, University of California San Francisco, San Francisco, CA, USA; 2 Diabetes Center, University of California San Francisco, San Francisco, CA, USA; 3 University of Georgia, Athens, GA, USA; 4 Department of Biophysics and Biochemistry, University of California San Francisco, San Francisco, CA, USA; † Swiss Institute for Experimental Cancer Research (ISREC), Swiss Federal Institute of Technology Lausanne (EPFL), Lausanne, Switzerland

**Keywords:** EGFR, Hb-egf, TGF-α, pericyte, cancer

## Abstract

Factors associated with tumor sensitivity to epidermal growth factor receptor (EGFR) inhibitors in the context of wild-type *EGFR* remain elusive. This study investigates the mechanistic basis of responsiveness to EGFR inhibitors in the RIP1-Tag2 (RT2) mouse model of pancreatic neuroendocrine tumorigenesis (PNET). Upon treatment of RT2 mice with EGFR inhibitors, PNET tumors harboring wild-type, nonamplified alleles of *Egfr* grow at a markedly reduced rate and display a significant increase in tumor cell apoptosis, as well as reduced neovascularization. The authors identify *Tgf*-α and *Hb-egf* as key limiting mediators of separable pathological functions of Egfr in neuroendocrine tumor progression: *Tgf*-α mutant tumors present with an elevated apoptotic index, whereas *Hb-egf* mutant lesions exhibit decreased angiogenic switching and neovascularization. This study not only associates Tgf-α and Hb-egf expression with wild-type *Egfr* oncogenicity but also ascribes the proangiogenic activity of Egfr in this tumor model to a novel mesenchymal Hb-egf/Egfr signaling axis, whereby endothelial and pericyte-derived Hb-egf activates Egfr specifically in tumor-associated perivascular cells, leading to increased pericyte coverage of the tumor endothelium and enhanced angiogenesis.

## Introduction

Members of the ErbB family of receptor tyrosine kinases, comprising ErbB1 (epidermal growth factor receptor [EGFR]), ErbB2 (Her2/Neu), ErbB3, and ErbB4, have pathological functions in a wide range of tumors, as they activate a variety of signaling pathways inside tumor and stromal cells to sustain proliferation, survival, angiogenesis, invasion, and metastasis.^1-3^ Signaling cascades that lie downstream of ErbB activation include the mitogen- activated protein (MAP) kinase and PI3K/Akt pathways.^4^ During normal development, ErbB signaling results from the binding of individual ErbB receptors with cognate ligands of the EGF family, which comprises epidermal growth factor (EGF), transforming growth factor–α (TGF-α), amphiregulin (AREG), epigen (EPGN), heparin-binding EGF-like growth factor (HB-EGF), betacellulin (BTC), epiregulin (EPR), and neuregulin 1 to 4 (NRG1-4). These ligands are highly redundant, have specific ErbB-binding patterns and affinities, and all except for the 4 NRG ligands bind and activate ErbB1/EGFR.^5^

In a wide range of human carcinomas (breast, pancreas, colon, head and neck, lung, etc.), EGFR and/or HER2 are significantly overexpressed, usually following gene amplification.^6^ Such gene amplification has been associated with a poor prognosis in a number of cancer types for both EGFR and HER2.^7^ However, while a correlation exists between the HER2 overexpression status in breast tumors and their sensitivity to HER2 inhibitors, such a correlation has failed to materialize in clinical trials involving EGFR inhibitors,^8^ leaving a gap in our understanding of tumor dependency on EGFR signaling. In non–small cell lung cancer (NSCLC), the presence of mutations in the EGFR kinase domain is a striking predictor of tumor sensitivity to EGFR inhibitors.^9-11^ In NSCLC patients, the frequency of *EGFR* mutations (~9% in non-Japanese patients^8^) is remarkably correlated with the objective clinical response (tumor shrinkage) rate observed in NSCLC patient cohorts (~10%),^12^ but it fails to account for the additional 30% of patients who present with stable disease following anti-EGFR treatment.^12^ In addition, data from two studies of NSCLC patients show that no *EGFR* mutation was detected in tumor samples from 6 of 31 patients presenting an objective clinical response to EGFR inhibitors.^9,11^ In metastatic colorectal cancer (mCRC) patients, the objective response rate to anti-EGFR antibody therapy of ~10% and the additional stable disease rate of ~30% cannot be predicted by chromosomal amplification of the *EGFR* locus observed in ~2% of mCRC patients^13^ and do not correlate with mutations in *EGFR*, observed in only 0.34% of CRC samples.^14-16^ These results collectively indicate that many patients with wild-type *EGFR* alleles respond to EGFR inhibitors, and research is ongoing regarding the factors that contribute to EGFR inhibitor tumor sensitivity, other than mutations in *EGFR* in NSCLC^12^ or chromosomal amplification of *EGFR* in colon cancer.^17^

It has long been known that ligands of the EGF family are overexpressed in a significant subset of solid tumors and are prognostic factors of poor disease outcome,^18^ suggesting that tumor progression may depend on the presence of these ligands. Given that *in vivo* overexpression of *Tgf*-α in mice is clearly pro-oncogenic in the context of wild-type *Egfr*, leading to pancreatic hyperplasia and mammary epithelial carcinogenesis,^19,20^ expression of EGF family ligands may be one of the keys to our understanding of tumor sensitivity to EGFR inhibitors. A preclinical study of subcutaneously transplanted human lung cancer cells supports this hypothesis, as TGF-α mediates tumor sensitivity to EGFR inhibitors in this system.^21^

The RIP1-Tag2 (RT2) mouse model of pancreatic neuroendocrine tumors (PNET)^22^ provides a well-studied *in vivo* platform in which to investigate the potential role of Egfr signaling in the stepwise progression of neoplastic lesions toward malignancy. The purification of betacellulin, a pan-ErbB EGF family ligand, from the conditioned media of RT2-derived cancer cells^23^ and the fact that a majority of human neuroendocrine tumors express phosphorylated Egfr^24^ suggested a possible involvement of ErbB signaling in multistage pancreatic neuroendocrine carcinogenesis. Neoplastic lesions in RT2 mice progress through several phenotypic transitions that are stereotypic to many forms of human carcinogenesis. About half of the 400 normal pancreatic islets become hyperplastic/dysplastic and begin to proliferate upon expression of the SV40 T antigen (Tag) oncogene. About 20% of the hyperproliferative islet lesions undergo angiogenic switching, and half of these angiogenic islets progress to the tumor stage, often beginning as encapsulated adenomas that progress to malignant invasive carcinomas.^25^

In this study, we demonstrate that RT2 PNET tumors engage the Egfr receptor *in vivo*, and while the tumors do not harbor chromosomal amplifications or mutations of the *Egfr* locus, they are nonetheless sensitive to pharmacological or genetic Egfr inactivation. Upon exposure to EGFR inhibitors, PNET tumors grow at a much reduced rate and present with a decreased neovasculature and an elevated apoptotic index. We ascribe the activation of two distinct pools of Egfr in PNET tumors (the first in cancer cells and the second in tumor-associated pericytes) to 2 EGF family ligands, Tgf-α and Hb-egf, which respectively mediate the antiapoptotic and proangiogenic activities of Egfr, revealing dual roles for Egfr signaling in this tumorigenesis pathway.

## Results

### Wild-type Egfr signaling contributes to the growth and neovascularization of PNET tumors

**Figure 1.**
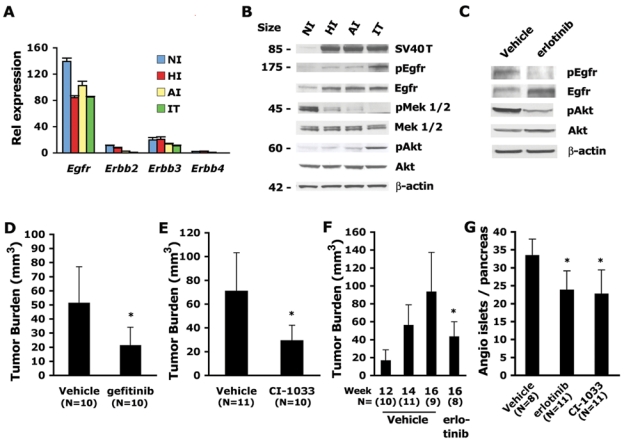
Egfr activity contributes to RT2 tumor growth and angiogenic switching. (**A**) Relative expression quantified by real-time quantitative PCR of *Erbb* family members in cDNAs derived from total RNA extracts of successive stages of pancreatic neuroendocrine tumorigenesis from RIP1-Tag2 (RT2) transgenic mice (NI = normal islets; HI = hyperplastic islets; AI = angiogenic islets; IT = islet tumors). Levels of mRNAs are expressed as a percentage of the *mGus* control mRNA. (**B**) Western blot analysis of protein extracts from successive stages of RT2-derived lesions. (**C**) Western blot analysis of protein extracts from RT2-derived tumors 4 h after treatment with a vehicle solution or erlotinib (80 mg/kg). (**D-F**) Comparison of the average tumor burden of RT2 mice treated daily with a control solution (vehicle) or with (**D**) gefitinib (80 mg/kg), (**E**) CI-1033 (80 mg/kg), both from 11.5 to 14.5 weeks of age, or with (**F**) erlotinib (80 mg/kg), from 12 to 16 weeks of age with an additional vehicle-treated time point at 14 weeks. (**G**) Comparison of the average number of hemorrhagic angiogenic islets per pancreas of RT2 mice treated daily with a vehicle solution or with erlotinib (80 mg/kg) or CI-1033 (80 mg/kg) from 6 to 9 weeks of age. (*N* = number of animals per treatment group). *P < 0.01.

To probe the potential role of EGFR signaling in PNET tumors of RT2 transgenic mice, we surveyed the expression profile of the ErbB family of receptors in each of the discrete stages of this tumorigenesis pathway. After isolating total RNA from pancreatic islets at different stages of disease progression (normal pancreatic islets, hyperplastic islets, angiogenic islets, and islet tumors), we measured the expression levels of *Egfr*, *Erbb2*, *Erbb3*, and *Erbb4* by quantitative real-time PCR (RT-PCR; Fig. 1A). Among Erbb receptors, *Erbb2* and *Erbb4* mRNA levels decreased with malignant progression and became barely detectable at the tumor stage. By contrast, *Egfr* and *Erbb3* were detected at all stages of tumor development, and the *Egfr* mRNA was the most prominently detected of the 4 Erbbs throughout the multistep pathway (Fig. 1A). We then surveyed the activation of Egfr and the concurrent activation of two signaling circuits that lie downstream of EGFR activation: mitogen-activated protein kinase (MAPK) and PI3K/Akt. Western blot analysis of protein extracts from the different RT2 stages revealed that Egfr phosphorylation increases during RT2 progression, as does Akt phosphorylation, reflecting the coincident activation of Egfr and the PI3K pathway (Fig. 1B). Surprisingly, pMek1/2, which indicates activation of the MAPK pathway, decreases steadily as RT2 lesions progress to malignancy (Fig. 1B). To assess the functional contribution of Egfr to Akt phosphorylation, we treated 14-week-old RT2 mice harboring advanced tumors with the EGFR inhibitor erlotinib for 4 days. Following erlotinib treatment, the phosphorylation of Egfr and Akt in tumor extracts was significantly reduced, indicating that Egfr activation contributes to Akt activation in these neoplastic lesions (Fig. 1C).

**Figure 2.**
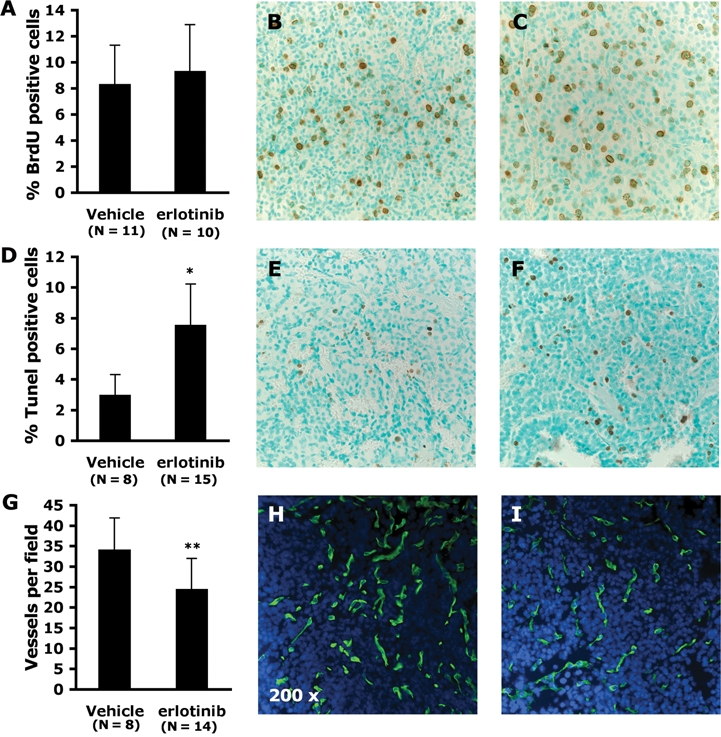
Phenotype of epidermal growth factor receptor (EGFR) inhibitor-treated pancreatic neuroendocrine tumorigenesis (PNET) tumors from RT2 mice. (**A**) Average percentage of dividing tumor cells (BrdU-positive) in vehicle- or erlotinib-treated mice following 1 week of treatment. (**B-C**) Representative micrographs of tumors from (**B**) vehicle- or (**C**) erlotinib-treated RT2 mice stained with an anti-BrdU antibody (200x). (**D**) Average percentage of apoptotic tumor cells (TdT-mediated dUTP-biotin nick end-labeling [TUNEL] positive) in tumors from vehicle- or erlotinib-treated mice following 1 week of treatment. (**E-F**) Representative micrographs of (**E**) vehicle- or (**F**) erlotinib-treated tumors stained with an anti-digoxigenin antibody following Tunel procedure (200x). (**G**) Average number of blood vessels per field (FITC-positive continuous segments) in vehicle- or erlotinib-treated mice following 1 week of treatment. (**H-I**) Representative micrographs of (**H**) vehicle- or (**I**) erlotinib-treated tumors that were collected following systemic perfusion with FITC-lectin to visualize the functional tumor vasculature (green); counterstaining with DAPI reveals the cellularity (blue) (200x). The panels are representative of 2 fields of tissue sections obtained from tumors in at least 4 treated RT2 mice. (*N* = number of independent tumors analyzed per treatment group). *P < 0.001. **P < 0.02.

To investigate the possibility that Egfr activation was involved in the pathobiology of these PNET tumors, we treated cohorts of RT2 mice with different EGFR inhibitors (gefitinib, CI-1033, erlotinib) for 3 to 4 weeks beginning at 11 to 12 weeks of age, a stage at which small adenoma are already present. Gefitinib and erlotinib are specific inhibitors of EGFR,^26^ whereas CI-1033, in addition to blocking EGFR, also inhibits the kinase activity of Erbb2 and Erbb4 (Erbb3 does not possess intrinsic kinase activity).^27^ All treated cohorts of RT2 mice displayed a striking 50% to 60% decrease in tumor burden (average total tumor volume per mouse) compared to vehicle-treated cohorts (Figs. 1 D-F). The growth of these tumors was not halted but was significantly slowed upon EGFR inhibitor treatment, as shown in Figure 1F (compare vehicle treated at week 12 to erlotinib treated at week 16). Moreover, we found that treating 6-week-old RT2 mice with EGFR inhibitors (erlotinib or CI-1033) for 3 weeks resulted in a ~30% decrease in the number of islets undergoing angiogenic switching (Fig. 1G), indicating that Egfr activity also contributes to this pathological transition. A detailed phenotypic analysis revealed that treated tumors displayed no decrease in tumor cell proliferation (assayed by BrdU incorporation) as compared to vehicle-treated tumors (Figs. 2 A-C) but had a markedly elevated apoptotic index (assayed by TdT-mediated dUTP-biotin nick end-labeling [TUNEL] staining) (Figs. 2 D-F) and significantly decreased vascular density (assayed by counting FITC-lectin perfused vessels) (Figs. 2 G-I).

In previous studies of human patients, sensitivity to EGFR inhibitors was correlated with activating mutations in the kinase domain of *EGFR* or with genomic amplification of the *EGFR* locus. We know from previous studies of chromosomal gains and losses in RT2 tumors that the mouse *Egfr* locus (chromosome 11-9.0 cM) is not amplified in RT2 malignancies.^28^ To determine whether mutations in the kinase domain could account for the sensitivity of these PNET tumors to EGFR inhibitors, we sequenced the kinase domain of *Egfr* from 6 independent tumors. As shown in Supplementary Figure S1, we detected no mutation of *Egfr* in these tumor DNA samples, although a silent polymorphism was found to be present in the BL6/J background of RT2 mice.

Thus, Egfr is increasingly phosphorylated as pancreatic islet neoplasias grow and progress toward a malignant state. Egfr contributes significantly to the downstream activation of Akt, a key cellular survival pathway, and Egfr activation promotes tumor growth, angiogenic switching, cancer cell survival, and tumor angiogenesis, as revealed by its pharmacological inhibition. Tumor sensitivity to EGFR inhibitors is observed despite the fact that these tumors harbor no mutation or amplification of the *Egfr* locus; moreover, comparable phenotypes observed following treatment with EGFR-specific inhibitors (gefitinib, erlotinib) or a pan-Erbb inhibitor (CI-1033) indicate that EGFR is likely the functionally predominant Erbb tyrosine kinase in this model of PNET tumorigenesis.

### Expression of Tgf-α and Hb-egf correlates with phenotypic transitions of PNET lesions

**Figure 3.**
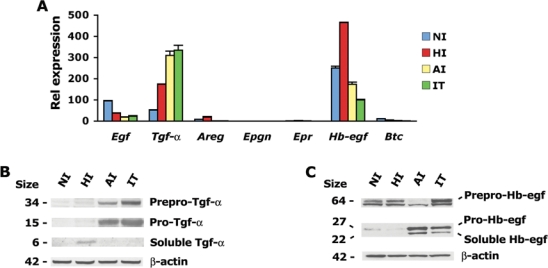
Dynamic expression and processing of Tgf-α and Hb-egf during multistep pancreatic neuroendocrine tumorigenesis (PNET). (**A**) Relative expression of *Egf* family members in cDNAs prepared from total RNA extracts of successive stages of neoplastic progression (NI = normal islets; HI = hyperplastic islets; AI = angiogenic islets; IT = islet tumors). Levels of mRNAs are expressed as a percentage of the *mGus* control mRNA. (**B**) Western blot analysis of the various forms of Tgf-α polypeptides in total protein extracts from successive stages of disease progression. (**C**) Analogous Western blot analysis of the various forms of Hb-egf polypeptides in total protein extracts from the successive disease stages.

Because wild-type Egfr is increasingly phosphorylated as RT2 lesions progress toward malignancy, we searched for candidate ligands that might mediate the increasing activation of Egfr by surveying the expression of all known genes of the EGF ligand family by quantitative RT-PCR during PNET tumorigenesis (Fig. 3A). This survey pointed toward two candidates, *Tgf*-α and *Hb-egf*, which were expressed at appreciably higher levels than the other ligand-encoding genes and also showed increased expression during one or more progressive disease stages. As shown in Figure 3A, levels of *Tgf*-α transcripts increase 6.5-fold between normal islets and the islet tumor stage, whereas *Hb-egf* expression increases ~2-fold at the hyperplastic stage and remains readily detectable at later stages of RT2. Interestingly, *Btc*, the EGF family ligand that was discovered in the conditioned medium of a RT2-derived cell line,^23^ was barely detectable in RT2 tumors, suggesting it is not a key *in vivo* mediator of Egfr activation in RT2. This was confirmed by crossbreeding RT2 mice into a homozygous *Btc*-null background^29^: mice lacking *Btc* did not present any defect in tumor growth or angiogenic switching (data not shown). The other ligands, including *Nrg-1*, -*2a*, -*2b*, *3*, and *4*, were expressed at very low levels or their expression levels decreased during tumor progression (data not shown) and were not considered further.

Next, we analyzed the protein expression profile of Tgf-α and Hb-egf, to visualize if these signaling peptides were processed into membrane-bound proforms or soluble forms, which are both bioactive.^30,31^ The low molecular weight soluble form of Tgf-α initially increases at the hyperplastic stage of RT2 progression before disappearing in later stages, whereas the membrane-bound proform of Tgf-α capable of stimulating EGFR phosphorylation,^32,33^ proliferation,^34^ and transformation^35^ in adjacent cells increases significantly at the angiogenic and tumor stages (>15-fold) of RT2 (Fig. 3B). In parallel, bioactive forms of Hb-egf (the 27-kD membrane-bound form and the 22-kD soluble form) increased dramatically at the angiogenic (>30-fold) and tumor stages (Fig. 3C).

Thus, the dynamic expression of bioactive Tgf-α and Hb-egf during RT2 progression was suggestive of their involvement at the angiogenic and tumor stages of RT2 carcinogenesis.

### Tgf-α enhances cancer cell survival and is required for the growth of PNET tumors in RT2 mice

**Figure 4.**
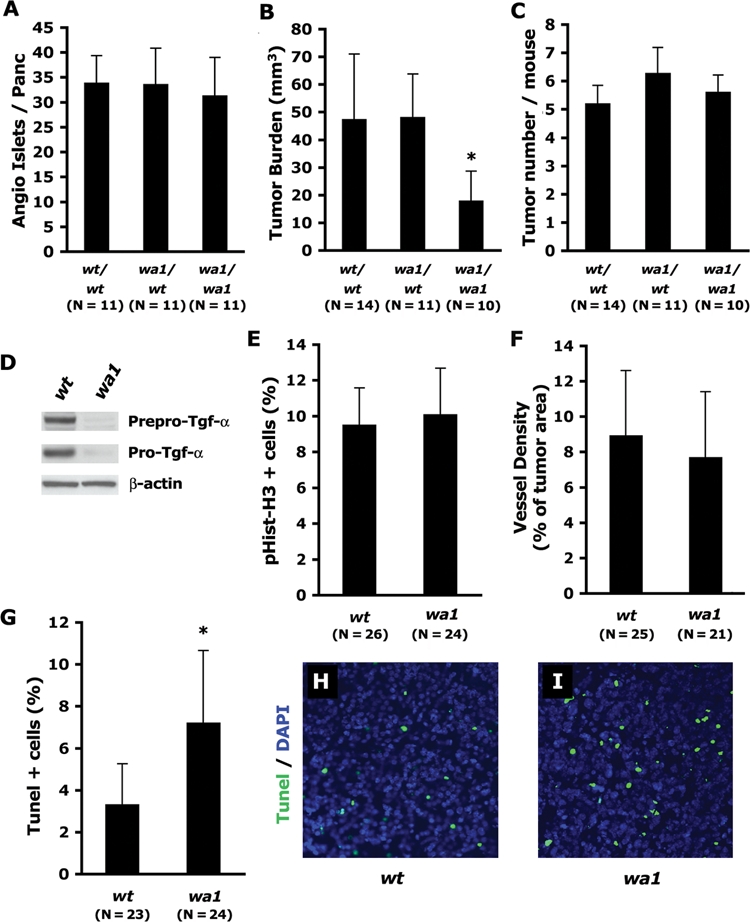
Tgf-α is required for tumor growth and cancer cell survival. (**A**) Comparison of the average number of hemorrhagic angiogenic islets dysplasias in wild-type (*wt/wt*), *waved-1* heterozygous (*wt/wa1*), and waved-1 homozygous (*wa1/wa1*) 9-week-old RT2 mice; *waved-1* is a loss-of-function allele of *Tgf*-α. (**B-C**) Comparison of the average (**B**) tumor burden or (**C**) tumor number in wild-type (*wt/wt*), *waved-1* heterozygous (*wt/wa1*), and waved-1 homozygous (*wa1/wa1*) 14-week-old RT2 mice. (**D**) Western blot analysis of Tgf-α protein expression in total protein extracts from pools of 5 wild-type or 5 *waved-1* mutant tumors. (**E**) Average percentage of dividing tumor cells (phospho-histone H3-positive) in wild-type or *waved-1* mutant tumors at 14 weeks of age. (**F**) Average vessel density (ratio of Meca-32 stained area to total section area) in wild-type or *waved-1* mutant tumors at 14 weeks of age. (**G**) Average percentage of apoptotic tumor cells (TdT-mediated dUTP-biotin nick end-labeling [TUNEL] positive) in wild-type or *waved-1* mutant tumors at 14 weeks of age. (**H-I**) Representative micrograph of a (**H**) wild-type or a (**I**) *waved-1* mutant tumor stained with anti-digoxigenin following the TUNEL procedure (green) and DAPI (blue) (200x); panels are representative of 12 wild-type and 16 *waved-1* RT2 tumors dissected from at least 5 independent mice of each genotype. (*N* = number of independent mice [**A-C**] or tumor fields [**D-F**] analyzed per genotype—1 to 2 fields per tumor). *P < 0.001.

Given the evident upregulation of Tgf-α during the PNET tumorigenesis pathway, we sought to assess its possible functional contribution by crossbreeding the RT2 transgenic mice with *waved-1* mutant mice harboring a mutant loss-of-function allele of *Tgf*-α that is characterized by the near extinction of Tgf-α expression.^36,37^ We surveyed the key parameters of tumor progression in cohorts of compound RT2: *Tgf*-α mutant mice of different ages, aiming to identify stages of disease progression that were affected by the loss of *Tgf*-α function. No difference was observed between wild-type and *waved-1* animals in the proportion of hyperproliferative islets at 7.5 weeks (data not shown) or in the number of hemorrhagic angiogenic islet dysplasias at 9 weeks (Fig. 4A). In contrast, average tumor burden was decreased in *Tgf*-α mutant mice (Fig. 4B) but not the average tumor number per mouse (Fig. 4C), indicating that Tgf-α enhances PNET tumor growth but not tumor initiation from the abundant pool of angiogenic islet precursor lesions. We confirmed by Western blot analysis that Tgf-α expression was abolished in *waved-1* tumor extracts (Fig. 4D). Then we dispelled the possibility that the decreased pathology observed in RT2, *wa-1/wa-1* mice could be due to preexisting developmental defects in pancreatic islets of homozygous *waved-1* mutants by comparing the morphology of pancreatic islets (Supplementary Figs. 2 A and B), the expression of insulin in pancreatic islets (Supplementary Figs. 2 C and D), and endocrine pancreatic function (via a glucose tolerance test) (Supplementary Fig. 2E) of wild-type versus *waved-1* homozygous animals. As no significant difference was detected in any of these parameters or in islet size (data not shown), we concluded that the decreased tumor growth observed in RT2, *wa-1/wa*-1 mutant mice did not result from a preexisting defect in endocrine pancreatic development but rather stemmed from the functional contribution of Tgf-α to neuroendocrine tumor growth.

To assess the pathological function of Tgf-α in PNET tumors, we compared the proliferation index (assayed by phospho-Histone H3 staining—Fig. 4E), blood vessel density (assayed by measuring areas of Meca-32 staining— Fig. 4F), and apoptotic index (assayed by TUNEL staining—Fig. 4G) of wild-type versus *waved-1* mutant tumors. We found that *Tgf*-α mutant tumors had elevated numbers of TUNEL-positive apoptotic cells compared with *Tgf*-α competent tumors (see representative panels—Fig. 4 H-I), whereas there were no changes in proliferation rate or tumor vascularity, indicating that Tgf-α functioned to enhance tumor growth by limiting the rate of cancer cell apoptosis.

### Hb-egf contributes to angiogenic switching and tumor neovascularization

**Figure 5.**
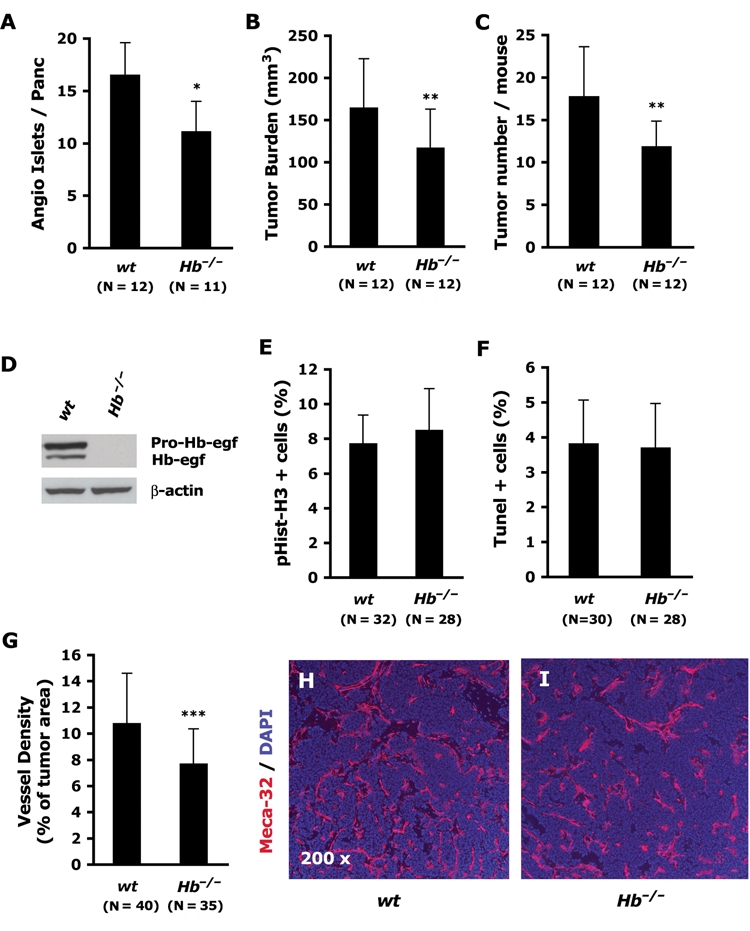
Hb-egf contributes to the angiogenic switch and neovascularization of PNET tumors. (**A**) Comparison of the average number of hemorrhagic angiogenic islets in wild-type (*wt*) and *Hb-egf* mutant (*Hb*) 9-week-old RT2 mice. (**B-C**) Comparison of the average (**B**) tumor burden or (**C**) tumor number in wild-type and *Hb-egf* mutant 14-week-old RT2 mice. (**D**) Western blot analysis of Hb-egf protein expression in total protein extracts from pools of 5 wild-type or 5 *Hb-egf* mutant tumors. (**E-F**) Average percentage of (**E**) dividing tumor cells (phospho-histone H3 positive) or (**F**) apoptotic cells (TdT-mediated dUTP-biotin nick end-labeling [TUNEL] positive) in wild-type or *Hb-egf* mutant tumors at 14 weeks of age. (**G**) Average vessel density (ratio of Meca-32 stained area to total section area) in wild-type or *Hb-egf* mutant tumors at 14 weeks of age. (**H-I**) Representative micrographs of (**H**) wild-type or (**I**) *Hb-egf* mutant RT2 tumors stained with Meca-32 (red) and DAPI (blue) (200x); panels are representative of 20 wild-type and 18 *Hb-egf* RT2 tumors dissected from at least 5 independent mice of each genotype. (*N* = number of independent mice [**A-C**] or tumor fields [**E-G**] analyzed per genotype; 1-2 fields per tumor.) *P < 0.001. **P < 0.01. ***P < 0.05.

Given the elevated level of Hb-egf protein expression observed at the angiogenic and tumor stages of disease progression, we sought to identify the functional contribution of this ligand to the pathology of both premalignant and tumor stages. We bred *Hb-egf* null mutant mice^29^ to RT2 transgenic animals, following extensive back-crossing of the *Hb-egf* allele into the C3HeB/FeJ genetic background (see Methods) because homozygous *Hb-egf* mutants were nonviable in a pure C57BL/6J background. We assessed the same histopathological parameters of tumor progression described previously, comparing cohorts of wild-type RT2 versus RT2, *Hb-egf*^*−/−*^ littermates at different stages of cancer progression. We found no difference in the proportion of BrdU-positive islets at 7.5 weeks of age (data not shown), indicating that Hb-egf is not involved in the onset of hyperproliferation that leads to hyperplastic/dysplastic (preangiogenic) islets. In contrast, the absence of Hb-egf affected the angiogenic islet and tumor phenotypes. There was a ~30% decrease in the average number of angiogenic islets in 9-week-old *Hb-egf* mutant mice (Fig. 5A), at the peak of angiogenic switching but before the formation of solid tumors. And in 14.5-week-old RT2, *Hb-egf*^*−/−*^ mutant mice, we observed both a ~30% decrease in the average total tumor load per mouse (tumor burden—Fig. 5B) and a similar decrease in the average number of tumors per mouse (Fig. 5C).

We performed a Western blot analysis of protein extracts from wild-type and *Hb-egf* mutant tumors and confirmed the depletion of the Hb-egf ligand in *Hb-egf* mutant tumors (Fig. 5D). We also verified that normal endocrine pancreatic development was normal in *Hb-egf* mutant mice to rule out the possibility that the observed tumor phenotypes could be secondary consequences of developmental defects occurring before the onset of tumorigenesis. The histology and morphology of islets of Langerhans were normal in *Hb-egf* mutant mice (Supplementary Figs. S3 A and B), the pattern of insulin staining was not affected in *Hb-egf* mutant pancreas (Supplementary Figs. S3 C and D), and *Hb-egf* mutant mice presented no defects in glucose tolerance, indicating normal pancreatic β-cells function (Supplementary Fig. S3E).

Having established that Hb-egf was not involved in normal islet development or homeostasis, we proceeded to analyze the phenotype of *Hb-egf* mutant tumors in detail. There was no evident difference in the proliferation and apoptotic indices between wild-type and *Hb-egf* mutant tumors (Figs. 5 E-F). There was, however, a 28% decrease in vessel density in *Hb-egf* mutant tumors (Fig. 5G—representative panels Figs. 5H-I). Together, these analyses indicate that Hb-egf is involved in regulating angiogenic switching in premalignant lesions and the growth and/or maintenance of the angiogenic vasculature in PNET tumors. The fact that (1) *Hb-egf* mutant tumors did not display defects in apoptosis or proliferation, (2) the number of tumors per *Hb-egf* mutant mouse was decreased as compared to wt mice, but (3) not the average size of individual tumors across the 2 mouse cohorts (10.22 mm^3^ in wt mice—*N* = 228 vs 9.69 mm^3^ in *Hb-egf* mutant mice—*N* = 143; *P* = 0.2283) collectively indicates that defects observed at the tumor stage may be secondary to defects in angiogenic switching observed at an earlier stage of disease progression (i.e., that *Hb-egf* mutant mice present a decreased tumor load because they develop fewer angiogenic precursor lesions, not because of a defect per se in tumor cell growth or apoptosis).

### Distinct pools of Egfr activity in PNET cancer cells and in perivascular cells

**Figure 6.**
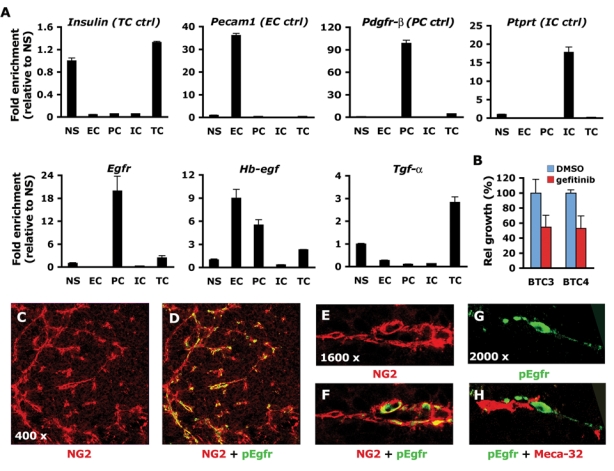
Distinct pools of Egfr activity in cancer cells and perivascular cells. (**A**) Expression of cell-type defining genes *m-Insulin1*, *Pecam1*, *Pdgfr*-β, *Ptprt*, and of *Egfr*, *Hb-egf*, and *Tgf*-α in sorted cells from RT2-derived PNET tumors (NS = nonsorted cells; EC = endothelial cells; PC = pericytes; IC = immune cells; TC = unlabeled tumor cells) relative to the expression detected in nonsorted cells (NS = 1). Gene expression in each cellular fraction was normalized to levels of *Cyclophilin*. (**B**) Comparison of the relative *in vitro* growth of 2 RT2 tumor-derived cancer cell lines (BTC3 and BTC4) following 3 days of treatment with DMSO or gefitinib 5 μM. (**C**) Anti-NG2 staining (red) reveals pericytes (200x). (**D**) Co-localization of NG2 (red) with phospho-EGFR Tyr1068 (pEgfr, green) (200x). (**E**) High magnification confocal localization reveals expression of phospho-Egfr (green) in pericytes expressing NG2 (red) (1600x). (**F**) High-magnification confocal localization of phospho-Egfr (green) and an endothelial specific marker (Meca-32, red) does not indicate Egfr activity in tumor endothelial cells (2,000x). (**C-H**) Micrographs are representative of multiple fields of more than 10 tumors from at least 3 independent RT2 mice.

To better understand the distinct histopathologic phenotypes observed in tumors of *Tgf*-α versus *Hb-egf* loss-of-function mutants and to relate them to the effects seen in EGFR inhibitor-treated tumors, we sought to identify the cell types expressing Egfr and its ligands inside tumors. To this end, we separated the different constituent cell types found in wild-type tumors by flow cytometry, by labeling endothelial cells with an anti-CD31 antibody, perivascular cells with an anti-PDGFR-β antibody, and immune cells with anti-GR1 and anti-CD11b antibodies. The various cellular fractions were represented as follows: tumor cells/other cells, 88.5%; immune cells, 2.8%; endothelial cells, 1.4%; pericytes, 1.8%; and gated-out, 5.5%. Total RNA isolated from these fluorescence-activated cell sorting (FACS)–sorted cell populations was converted to cDNA and analyzed by quantitative RT-PCR to assess the expression patterns of *Tgf*-α, *Hb-egf*, and *Egfr* alongside cell-type specific control genes that establish the purity of the sorted cell populations. Relative expression levels of the control genes indicated that the FACS procedure yielded very pure cellular fractions, with minimal cross-contamination of the specific markers in the other sorted populations (Fig. 6A—TC, EC, PC, and IC control panels). The analysis reveals that *Egfr* is expressed in cancer cells, is not detected in tumor endothelial cells or infiltrating immune cells, but, surprisingly, is highly enriched in PDGFR-β positive cells that predominantly comprise endothelium-associated perivascular cells in the PNET tumors of RT2 mice.^38^ *Hb-egf* is highly enriched in tumor endothelial cells and perivascular cells and, to a lesser extent in cancer cells, whereas *Tgf*-α appears exclusively enriched in the unlabeled cancer cell fraction (Fig. 6A).

The presence of 2 distinct pools of *Egfr* mRNA, one in cancer cells and one in perivascular cells, led us to explore Egfr function in both of these cellular compartments. We first confirmed that Egfr is functionally activated and required inside the cancer cells, as we were able to detect the phosphorylated form of Egfr (pEgfr) in cancer cells *in vivo* (Supplementary Fig. S4A). We then subjected RT2 tumor-derived cancer cell lines (BTC3 and BTC4) to treatment with the *gefitinib* EGFR inhibitor, after profiling of Egf family ligands and Erbb receptors in these BTC cell lines revealed that they express the same family members *in vitro* (*Egfr*, *Tgf*-α, and *Hb*-*egf*) as the cognate tumors do *in vivo* (Supplementary Figs. S4 B and C). We found that *gefitinib* has a significant impact on the growth of BTC lines *in vitro* (Fig. 6B) as well as on tumor growth *in vivo* (Fig. 1F), with the cell culture result substantiating the conclusion that Egfr signaling is involved in stimulating the cancer cells in a cell autonomous manner, consistent with the phenotype of *Tgf*-α mutant RT2 mice.

We then explored the possible function of Egfr inside tumor vessel-associated pericytes. First, we were able to co-localize pEgfr with NG2, a pericyte marker, indicating that Egfr is phosphorylated and activated in a subset of tumor pericytes *in vivo* (Figs. 6 C-F). Given the close proximity of pericytes with endothelial cells, we sought to assess the possibility that pEgfr was actually expressed in endothelial cells and mistakenly co-localized with pericytes. Therefore, we stained tumor sections for pEgfr and the Meca-32 endothelial cell marker, which revealed that pEgfr-positive cells and endothelial cells are adjacent but clearly distinct cell types (Figs. 6 G-H).

These analyses reveal that two cellular compartments express active Egfr inside these PNET tumors: cancer cells and pericytes. Similarly, ligand expression is compartmentalized: Tgf-α is exclusively parenchymal, whereas Hb-egf is expressed by the tumor parenchyma as well as the tumor stroma. These observations, coupled with the distinct and separable phenotypes observed in the *Tgf*-α and *Hb-egf* mutants, implicate two parallel Egfr intercellular signaling circuits in these PNET tumors: one circuit involves membrane-tethered Tgf-α that signals in autocrine/juxtacrine fashion to Egfr expressed as well on cancer cells, thereby enhancing cancer cell survival, while the second circuit involves Hb-egf expressed in multiple cell types, which signals to Egfr expressed on pericytes of the tumor vasculature.

### Hb-egf activates Egfr in pericytes and enhances pericytes coverage of the tumor endothelium

Because Hb-egf is a potent mitogen for smooth muscle cells and is able to trigger smooth muscle cell migration toward endothelial cells *in vitro*,^39,40^ we pursued the hypothesis that Hb-egf activates Egfr signaling inside perivascular cells and stimulates pericyte coverage of the tumor vasculature *in vivo*. We tested this hypothesis by staining the tumor vasculature with an endothelial marker (Meca-32) and a mature pericyte marker (Desmin) to determine the relative pericytic coverage of the tumor vasculature comparing control tumors, erlotinib-treated tumors, and *Hb-egf* mutant tumors.

**Figure 7.**
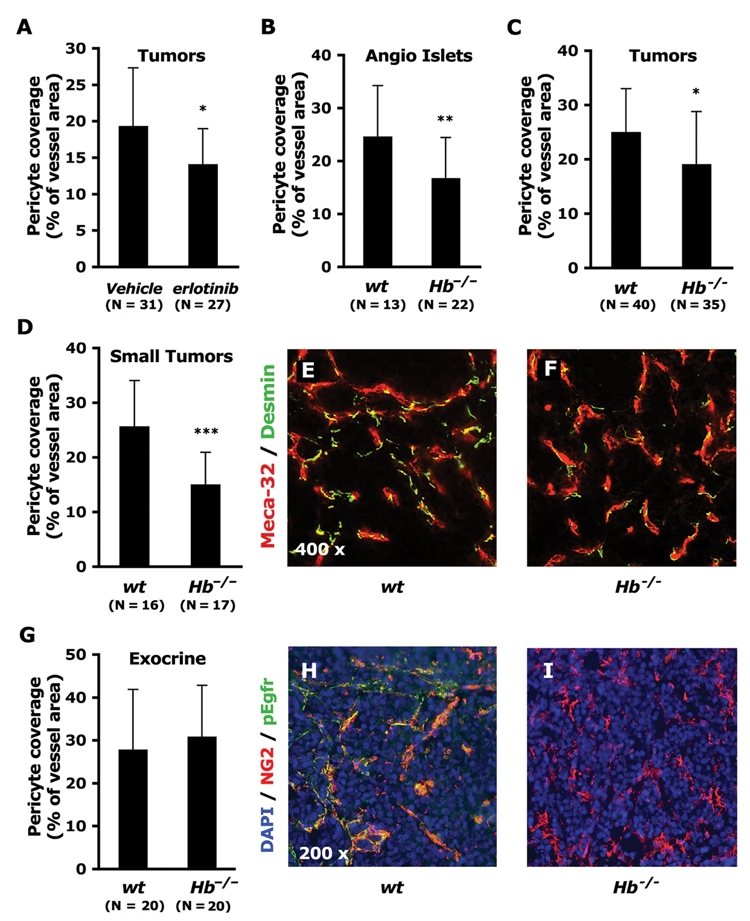
Contributions of Hb-egf and Egfr to pericyte coverage of the tumor neovasculature. (**A-D, G**) Pericyte coverage of the endothelium as a percentage of total vessel area in different stages and genetic contexts. (**A**) Comparison of vehicle- and erlotinib-treated tumors from 14-week-old RT2 mice. (**B**) Comparison of angiogenic islets from 9-week-old wild-type versus *Hb-egf* mutant RT2 mice. (**C**) Comparison of wild-type versus *Hb-egf* mutant tumors from 14-week-old RT2 mice. (**D**) Comparison of small wild-type and Hb-egf mutant tumors (ø < 3 mm) from 14-week-old RT2 mice. (**G**) Comparison of wild-type and mutant exocrine pancreas. (**E-F**) Representative staining of angiogenic microvessels (with Meca-32; red) and pericytes (with Desmin; green) (400x) in small (**E**) wild-type and (**F**) *Hb-egf* mutant tumors. (*N* = number of fields of tumors or angiogenic islet analyzed per genotype or treatment group; 1-2 fields per lesion analyzed.) (**H-I**) Representative staining of a pericyte marker (NG2; red) and activated phospho-Egfr (green) in tumors from (**H**) wild-type or (**I**) *Hb-egf* mutant RT2 mice. Micrographs are representative of several fields of more than 10 RT2 tumors from at least 3 independent mice of each genotype. *P < 0.05. **P < 0.01. ***P < 0.001.

Congruent with this hypothesis, tumors treated with an EGFR inhibitor displayed a significant ~27% decrease in mature pericyte coverage of the tumor endothelium (Fig. 7A). Pericyte coverage was decreased by ~32% in *Hb-egf* mutant angiogenic islets (Fig. 7B), by ~24% in large tumors (Fig. 7C), and by ~41% in small tumors (ø ≤ 3 mm—Fig. 7D) from *Hb-egf* mutant RT2 mice (representative tumor panels from each genotype, Figs. 7 E-F). Analysis of the unaffected exocrine compartment of the tumor-bearing pancreas of *RT2*, *Hb-egf*^*−/−*^ mice revealed no effect on vessel density (data not shown) or on pericyte coverage of the normal tissue vasculature (Fig. 7G), indicating that the effect on pericyte coverage is specific to the tumor vasculature. Moreover, *Tgf*-α mutant tumors showed no decrease in vessel density (Fig. 4F) or in pericyte coverage (data not shown), demonstrating that EGF family ligands are limiting for specific and distinct pathological functions *in vivo*.

Finally, we observed the near-complete disappearance of perivascular phospho-Egfr staining in *Hb-egf* mutant tumors (Figs. 7 H and I), thus further supporting our hypothesis that Hb-egf is the key limiting ligand involved in Egfr signaling inside perivascular cells.

### Mesenchymal Hb-egf mediates Egfr activation and functions in tumor-associated pericytes

**Figure 8.**
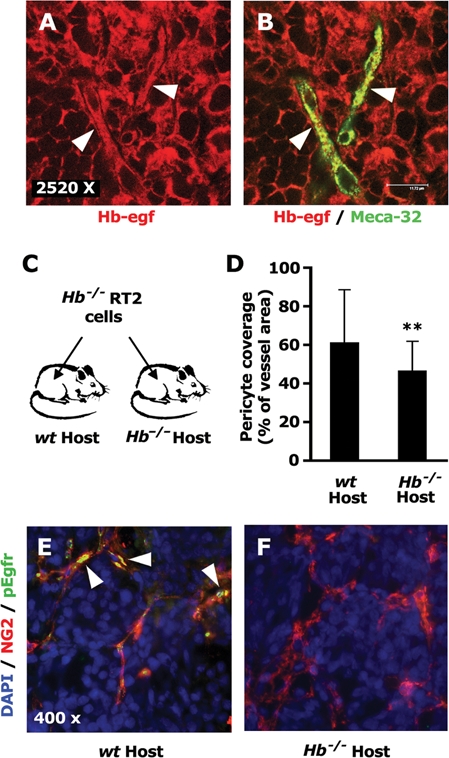
Mesenchymal-derived Hb-egf activates Egfr inside tumor pericytes. (**A-B**) Co-staining of Hb-egf staining (red) and Meca-32 (endothelial cell marker; green) by confocal microscopy (2,520x) reveals both cancer cell expression (red) and endothelial cell co-localization (yellow) of Hb-egf. (**C**) Schematic description of the orthotopic transplant experiment aimed at assessing the contribution of mesenchymal-derived Hb-egf to the angiogenic phenotype of pancreatic neuroendocrine tumorigenesis (PNET) tumors. (**D**) Comparison of pericyte coverage of the tumor neovasculature in wild-type (wt) versus Hb-egf mutant (Hb−/−) hosts. **P < 0.0005. (Number of tumors analyzed for each host genotype: N^wt^ = 11, N^Hb^ = 12.) (**E-F**) Representative staining of tumors for nuclei (DAPI; blue), NG2 (pericyte marker; red), and pEgfr (green) illustrating that the readily detectable pEgfr signal localized to pericytes in wild-type hosts (white arrows) (**E**) disappears in *Hb-egf* mutant hosts (**F**). pEgfr can be detected in cancer cells but at many-fold lower levels than in perivascular cells (see Supplementary Fig. S4). Micrographs are representative of 2 fields of 12 and 11 tumors obtained from wt or *Hb-egf* mutant hosts, respectively.

Given that Hb-egf is expressed in tumor cells as well as in tumor endothelial cells (Figs. 8 A and B) and pericytes (Fig. 6A), we sought to establish which reservoir of Hb-egf was responsible for mediating the proangiogenic activity of Egfr. To this end, we obtained an *Hb-egf* null cancer cell line from an *Hb-egf* mutant RT2-derived tumor and implanted it orthotopically in the pancreas of 8 syngeneic wild-type mice or 8 *Hb-egf* mutant mice (see schematic in Fig. 8C). This *Hb-egf* mutant cell line produced tumors in both genetic backgrounds, but the pericyte coverage of the tumor neovasculature was decreased by ~24% in tumors recovered from *Hb-egf* mutant hosts compared to tumors recovered from wild-type hosts (Fig. 8D). The vascular density of tumors grown in the *Hb-egf* mutant hosts was also decreased by ~20% compared to tumors grown in wild-type hosts (data not shown). Moreover, perivascular Egfr phosphorylation, while less prominent in the orthotopically transplanted tumors than in spontaneous tumors arising in RT2 mice, was nonetheless almost entirely eliminated in orthotopic tumors grown in *Hb-egf* mutant hosts (Figs. 8 E and F).

Together, these results indicate that the mesenchymal pool of Hb-egf retained in wild-type syngeneic hosts is responsible for the *in vivo* activation of Egfr signaling in perivascular cells and for the enhancement of tumor angiogenesis and pericyte coverage of the tumor endothelium.

## Discussion

Successful anticancer therapy with EGFR inhibitors is currently predicated on uncovering genetic and epigenetic factors that identify tumors addicted to Egfr signaling for their malignant phenotype. To date, mutations in the EGFR kinase domain^12^ and chromosomal amplification of the EGFR receptor^17^ are the only clinically validated genetic factors with positive predictive value of EGFR inhibitor sensitivity, whereas the presence of a Kras mutation is a genetic factor with negative predictive value.^41^ The low frequency of objective clinical responses in patients carrying wild-type, nonamplified alleles of *EGFR* has raised the legitimate question as to whether wild-type *EGFR* is even oncogenic.^8^ However, clinical responders with wild-type *EGFR* do exist, and the frequency of patients displaying stable disease in response to EGFR inhibitors far exceeds the frequency of *EGFR* kinase domain mutations.^12^

Our study in a mouse model of pancreatic neuroendocrine carcinogenesis demonstrates that tumors harboring wild-type diploid alleles of *Egfr* can be sensitive to EGFR inhibitors, and thus wild-type *Egfr* is tumor promoting in some tumor types. Notably, however, the significant slowing of tumor growth we observe is not regarded as an objective response in a clinical setting where tumor shrinkage is expected. Nonetheless, our results, combined with observations that (1) EGFR levels correlate with the malignant grade of endocrine tumors,^42^ (2) EGFR inhibitors increase apoptosis in a human PNET cell line,^42^ and (3) phosphorylated EGFR is detected in most human neuroendocrine tumors,^24^ suggest that some PNET patients may benefit from anti-EGFR therapy. Our results also argue that the detection of phosphorylated EGFR in conjunction with upregulated expression of an EGF family ligand, particularly TGF-α, a known prognostic factor of poor disease outcome in numerous cancer types,^18^ may prove an informative criterion of patient stratification when evaluating response to anti-EGFR therapy. Furthermore, *TGF*-α expression was strongly correlated with EGFR inhibitor sensitivity and apoptosis induction upon EGFR inhibitor treatment in a panel of 42 NSCLC cell lines, only 2 of which carried *EGFR* mutations.^43^ Interestingly, while we anticipated that upregulated TGF-α in the cancer cells would act as a mitogenic growth factor via autocrine/juxtacrine signaling to Egfr expressed in those cancer cells, both genetic and pharmacological interference with this signaling axis caused an increase in the frequency of apoptosis, not a reduction in cancer cell proliferation. The basis both of mitogenic stimulation in these PNET tumors and the mechanisms by which TGF-α acts as a survival factor warrants future investigation.

### Hb-egf activates Egfr in pericytes of the tumor vasculature

In this study, we observed a reproducible decrease in the microvascular density of tumors treated with EGFR inhibitors or genetically depleted of the Hb-egf ligand. Studies to date have described 2 proangiogenic mechanisms for EGFR signaling: first, some tumor cells induce critical angiogenic factors, such as VEGF-A, in an EGFR-dependent manner^44,45^; second, direct EGFR activation inside endothelial cells has been reported in several subcutaneous and orthotopic xenograft tumor models^46,47^ and found to support endothelial cell survival and tumor growth.^48^ By contrast, in the spontaneous RT2 model of mouse PNET as well as in one anecdotal human PNET sample (data not shown), we found that endothelial cells did not express EGFR; in addition, we did not detect a decrease in *Vegf-a* expression following gefitinib treatment (data not shown). Instead, we uncovered a novel *in vivo* mechanism whereby Hb-egf → Egfr signaling contributes to tumor angiogenesis: Egfr is directly activated inside tumor-associated pericytes, a mesenchymal cell type physically associated with microvascular endothelial cells, which supports endothelial cell function and integrity.^49^ We observe that Hb-egf secreted by cell types of the tumor stroma, either the pericytes themselves or the tumor endothelial cells, contributes to the pericyte coverage of the tumor endothelium. Our results corroborate an earlier *in vitro* study in which HUVEC endothelial cells were shown to express HB-EGF and promote the migration, in a trans-well migration assay, of EGFR-positive smooth muscle cells toward endothelial cells in an HB-EGF/EGFR-dependent manner,^40^ as well as an *in vivo* study from the same group describing decreased pericyte coverage of the tumor microvasculature in transplanted tumors following *gefitinib* treatment.^50^

Two questions relating to the interplay between pericytes and endothelial cells *in vivo* arise from our work: first, what mechanism underlies the decrease in Desmin-positive pericyte coverage following Egfr signaling inhibition (consequent to inhibitor treatment or genetic ablation of Hb-egf), and second, how does a reduction in pericyte coverage translate into a decreased density of the tumor microvasculature?

In regard to the first question, induction of Desmin expression appears to define the final step of pericyte differentiation, which involves intimate interaction with endothelial cells.^38^ Perivascular cells expressing other markers, such as Pdgfr-β (the earliest marker of pericyte differentiation) or NG2 plus α-SMA, define pools of partially differentiated perivascular precursor cells from which fully differentiated Desmin-positive cells eventually emerge.^38^ Following treatment with *erlotinib*, or in *Hb-egf* mutant lesions, we observe a more pronounced reduction in perivascular coverage by fully differentiated Desmin- positive cells as compared to NG2-positive precursor (~30% decrease in Desmin-positive cell coverage versus ~15% decrease in NG2-positive cell coverage—Figs. 7 C and D and data not shown). These results suggest that, when Egfr signaling is interrupted in perivascular precursor cells, as evidenced by the loss of pEgfr staining in the precursor NG2-positive pericytes of *Hb-egf* mutant tumors (Figs. 7 H and I), either those partially differentiated Desmin-negative pericyte precursors do not mature into fully differentiated Desmin-positive pericytes in the first place or such mature Desmin-positive pericytes are not stably maintained in association with the tumor vasculature.

As to how a pericyte coverage defect translates into a microvessel density decrease, it is established that interruption of endothelial/pericyte interaction with Pdgfr-β inhibitors indirectly affects the tumor microvasculature by catalyzing endothelial cell apoptosis^38^ and sensitizes the endothelium to additional stresses such as low-dose metronomic chemotherapy^51^ or Vegf receptor inhibition.^52^ This indirect effect on the tumor endothelium likely stems from the fact that pericytes produce a number of growth factors that support endothelial cell growth and survival.^49^ Much like with PDGFR inhibitors, EGFR inhibitors do not affect pericyte coverage or the homeostasis of normal tissue vessels, suggesting that other mechanisms preserve pericyte coverage in static, nonangiogenic endothelium.

In conclusion, this study (1) adds new support to the link between the expression of EGF family growth factors and tumor sensitivity to EGFR inhibitors in the context of wild-type *EGFR*; (2) demonstrates that EGF family ligands are not functionally redundant during tumorigenesis but play simultaneous, specific, and discrete pathological functions inside different cellular compartments of the same tumor; and (3) describes a novel *in vivo* proangiogenic mechanism driven by Egfr signaling inside pericytes and mediated by mesenchyme-derived Hb-egf. Finally, the parallel signaling axes we describe suggest that in some contexts, EGFR inhibitors may show combinatorial benefit with VEGF pathway inhibitors, whereby the EGFR inhibitor both impairs cancer cell survival and reduces pericyte coverage, with the latter rendering the tumor endothelium more sensitive to VEGFR inhibition.

## Materials and Methods

### Mouse Strains and Genotyping

All mouse strains were previously described.^22,29,36,53^ The *waved-1* mice (B6.Cg-Tgfa^wa1^/J strain) were obtained from Jackson Laboratory (Bar Harbor, ME) and were maintained in a pure C57BL/6J background; *Hb-egf* mutant mice were backcrossed for 10 generations in the C3HeB/FeJ background (we used this background because *Hb-egf* loss of function is nonviable in a pure C57BL/6J background). The RT2 transgene has been backcrossed for multiple generations (*N* > 60) in a pure C57BL/6J or a pure C3HeB/FeJ background. To genotype *waved-1* mice, we distinguished heterozygous from homozygous mice based on the wavy hair phenotype. Wild-type *Hb-egf* genotyping: *HB3* primer ccaggtataaataggacatttgagga and *HB4* primer ttgcaggaagac tgtgtcac (~400-bp band). Mutant *Hb-egf* genotyping: *HB4* primer and *ND2* primer tgctctttactgaaggctctttac at 0.5 μM (~300-bp band). All studies were conducted in compliance with University of California Institutional Animal Care and Use Committee (IACUC) guidelines.

### Mouse Treatment with EGFR Inhibitors

Compounds were obtained from commercial sources, reduced to a powder, and resuspended in a vehicle solution (0.5% carboxymethyl cellulose, 0.4% Tween-80, 0.9% NaCl) and orally delivered to mice in once-daily gavages at the following dosage: gefitinib (80 mg/kg), erlotinib (80 mg/kg), and CI-1033 (80 mg/kg). Mice were treated from 11 to 14 weeks of age in intervention trials, 6 to 9 weeks of age in prevention trials, and 12 to 16 weeks of age in regression trials. Mice were sacrificed 2 to 4 hours after the last oral delivery of drugs.

### Orthotopic Transplant Experiment

The pancreases of 8 syngeneic (C3H/HeJ) wild-type hosts (4 males and 4 females) obtained from Jackson Laboratory and of 8 *Hb-egf* mutant hosts (4 males and 4 females) were injected orthotopically with 250,000 BTC *Hb*^*−/−*^ cells resuspended in 50 μL of phosphate-buffered saline (PBS)/Growth Factor Reduced Matrigel™ (1:1) (#356231, BD Biosciences, Franklin Lakes, NJ) using a 28G1/2-gauge insulin syringe (BD #329461). After 11 weeks, following the death of a control animal injected with wild-type BTC cells, tumors were recovered from these mice and analyzed.

### Isolation of Pancreatic Islets

Islets from 10-week-old wild-type C57BL/6J mice were pooled to generate normal islet (NI) extracts. White islets from 6- to 8-week-old RT2 mice were used to generate hyperplastic islets (HI) extracts. Red islets from 6- to 8-week-old RT2 mice were used to generate angiogenic islet (AI) extracts. Islet tumor (IT) extracts were generated from islet tumors dissected from 14-week-old mice. The method for isolating islets of Langerhans from murine pancreas was previously described.^54^

### Quantitative PCR

Total RNA was prepared using RNeasy Mini™ (Qiagen, Valencia, CA) following the manufacturer’s recommendations. DNAse treatment and RNA cleanup were performed with the DNA-Free RNA Kit™ (Zymo Research, Orange, CA). cDNA synthesis was performed using qScript™ cDNA supermix (Quanta BioScience, Gaithersburg, MD). Quantitative real-time PCR was performed using the following TaqMan® assays (Applied Biosystems, Foster City, CA) or specifically designed assays: Mm00433023_m1 (*Egfr/Erbb1*), Mm00658541_m1 (*Erbb2*), For: cgggacccaccaaggtatc/Rev:ttggtgctcagagcagatgg/Probe:fam-tcatcaagagagcgagtgggcctgg-bhq1 (*Erbb3*), For: gctgctcaggaccaaaggac/Rev:agtaacgcaggctccactgtc/Probe:fam-ctgactgctttgcctgcatgaacttca-bhq1 (*Erbb4*), Mm00438696_m1 (*Egf*), Mm00437583_m1 (*Areg*), Mm00504344_m1 (*Epg*), Mm00446231_m1 (*Tgfa*), Mm00514794_m1 (*Epr*), Mm00432137_m1 (*Btc*), Mm00439307_m1 (*Hb-egf/Dtr*), Mm00626552_m1 (*Nrg-1*), For:aatggaggcgtgtgctactaca/Rev:ccgaagaatccgtttggaca/Probe:fam-cgaaggcatcaaccaactctcctgca-bhq1 (*Nrg-2a*), For: tggaggcgtgtgctactacatc/Rev:cccggtgtatcccacagg/Probe: fam-aaggcatcaaccaactctcctgcaagtg-bhq1 (*Nrg-2b*), Mm004- 35367_m1 (*Nrg-3*), Mm00446254_m1 (*Nrg-4*), Hs01076092_m1 (Human EGFR), Hs00961131_m1 (Human HBEGF), Hs00177401_m1 (Human TGF-α), For:ctcatctggaatttcgccga/Rev:ggcgagtgaagatccccttc/Probe:fam-cgaaccagtcaccgctgagagtaatcg-bhq1 (*mGus*), Mm00435546_m1 (*PDGFR*-β), Mm00476702_m1 (*Pecam1/CD31*), and Mm00448463_m1 (*Ptprt/CD45*). The *IDT* assays were obtained from Integrated DNA Technologies (Coralville, IA). All assays amplified a specific amplicon from a testis, brain, or embryonic mouse cDNA library (#10667012, Invitrogen, Carlsbad, CA). qPCR reactions were performed on an ABI7900HT Sequence Detection System. Ct values were determined and subtracted to obtain the ΔCt [ΔCt = Ct (test locus) – Ct (control locus)]. Relative fold difference was calculated as 2Δ^Ct^ × 100.

### Western Blot Analysis

The following antibodies were used: goat anti-TGF-α (0.2 μg/mL [R&D Systems, Minneapolis, MN, #AF-239-NA]), goat anti-HB-EGF (0.2 μg/mL [R&D Systems #AF-259-NA]), rabbit anti-SV40 large T antigen (1:10,000),^55^ rabbit anti-EGFR (1:200 [Santa Cruz Biotechnology, Santa Cruz, CA, #sc-03]), mouse anti-phospho-EGFR (1:1,000 [Cell Signaling, Danvers, MA, #2236]), rabbit anti-MEK1/2 (1:2,000 [Cell Signaling #9122]), rabbit anti-phospho-MEK1/2 (1:1,000 [Cell Signaling #9154]), rabbit anti-Akt (1:2,000 [Cell Signaling #9272]), rabbit anti-phospho-Akt (1:1,000 [Cell Signaling #9271]), mouse anti-β-actin (1:10,000 [Sigma-Aldrich, ST. Louis, MO, #A-5441]). Relative band quantifications were obtained using Photoshop 7.0 software.

### Histological Analysis, Immunochemistry, and Immunofluorescence

All tumors analyzed were considered “islet tumors” stage lesions with a diameter >1 mm. Tumors were snap-frozen in liquid nitrogen upon dissection and arrayed in OCT (Tissue-Tek #4583). Sections 10 μm thick were briefly fixed in ice-cold acetone or paraformaldehyde 3.2% for 10 min or nonfixed depending on the antibody used. Sections stained with the anti-pEGFR antibodies were subjected to an antigen-unmasking procedure (Vector Laboratories, Burlingame, CA, #H-3300). For use of mouse primary antibodies, slides were incubated with MOM™ kit reagent (Vector Laboratories #BMK-2202). Primary antibodies were applied overnight at 4°C. The following antibodies were used: rat anti–Pan Endothelial Cell Antigen antibody (Meca-32 clone, BD/Pharmingen, San Diego, CA, #553849), goat anti-HB-EGF (10 μg/mL [R&D Systems #AF-259-NA]), mouse anti-phospho-EGFR1-Tyr1068 (1:100 [Cell Signaling #2236]), rabbit anti-phospho-EGFR2-Tyr845 and Tyr992 (1:100 [Cell Signaling #2231, #2235]), rabbit anti-NG2 (1:200 [Millipore, Billerica, MA, #AB5320]), rabbit anti-glucagon (1:4,000 [Millipore #4030-01F]), rat anti-PDGFR-β (1:100 [eBioscience, San Diego, CA, #14-1402-81]), mouse anti-Desmin (1:100 [Dako, Glostrup, Denmark, #M0760]), and mouse antihuman CD34 (1:100 [BD Pharmingen, San Diego, CA, #555820]). Fluorochrome-conjugated antibodies were used as secondary antibodies (1:200 [Jackson ImmunoResearch, West Grove, PA]). Images of H&E- and DAB-stained sections were obtained with a Zeiss AxioImager brightfield microscope, fluorescent images were obtained with a Zeiss AxioScope2 widefield microscope, and confocal images were obtained with a Leica SL confocal microscope.

### Vessel Density and Vessel Coverage Analysis

FITC-lectin labeling of the vasculature was previously described.^56^ FITC-positive vessels were counted manually, or vessel area (labeled with Meca-32 antibody) and pericyte area (labeled with anti-Desmin antibody) were analyzed and percentage of pixel overlap measured with the MetaMorph® software (angiogenesis application). For each condition or genotype, 2 pictures were taken for each tumor lesion analyzed, one at the center and one at the periphery of the tumor. For vessel density analysis, pictures were taken at 200x magnification. Each picture represents ~0.4 mm^2^ or about ~8% of the average surface of RT2 tumors (2.5 mm in ø ~ 5 mm^2^). For pericyte coverage analysis, pictures were taken at 400x magnification, and each picture represents ~0.1 mm^2^ or about ~2% of the average surface of RT2 tumors.

### Apoptosis and Proliferation Assays

TUNEL and BrdU detection assays were performed as previously described.^57^ A rabbit anti-Phospho-Histone H3 antibody (1:100 [Upstate/Millipore #06-570]) was also used to detect cell divisions *in vivo*. Positive nuclei were counted using the MetaMorph® software (cell count application).

### *In Vitro* BTC Cell Growth Assay

In total, 300,000 beta-TC3 or beta-TC4 cells were plated on day 0 in 6-well culture dishes. A gefitinib solution (5 mM gefitinib dissolved in DMSO) was diluted 1,000x in the Dulbecco’s modified Eagle’s medium (DMEM) high-glucose cell culture media (Gibco, Carlsbad, CA, #11971), supplemented with 0.5% fetal calf serum. DMSO diluted 1,000x was used as control. The media were replaced every 24 h. After 72 h, cells were counted.

### Flow Cytometry

Tumors were excised from 14-week-old RT2 mice; inflammatory cells, endothelial cells, pericytes, and tumor cells were collected by FACS sorting as previously described.^38,52^

## Supplementary Material

Supplementary Material
