# Supplementary material for: Differential Contribution to Neuroendocrine Tumorigenesis of Parallel Egfr Signaling in Cancer Cells and Pericytes
Source: Genes Cancer. 2010 Feb;1(2):125–41. doi: 10.1177/1947601909358722 (PMC2958675; doi:10.1177/1947601909358722)
Supplement: Supplementary Material [file supp_1_2_125__index.html]

Differential Contribution to Neuroendocrine Tumorigenesis of Parallel Egfr Signaling in Cancer Cells and Pericytes — Supplementary Material 

# Differential Contribution to Neuroendocrine Tumorigenesis of Parallel Egfr Signaling in Cancer Cells and Pericytes

## Supplementary Material

**Files in this Data Supplement:**

- Figures S1-S4
