# Supplementary material for: Differential Contribution to Neuroendocrine Tumorigenesis of Parallel Egfr Signaling in Cancer Cells and Pericytes
Source: Genes Cancer. 2010 Feb;1(2):125–41. doi: 10.1177/1947601909358722 (PMC2958675; doi:10.1177/1947601909358722)
Supplement: Supplementary Material [file supp_1947601909358722_02GANC-Nolan-Stevaux_DS_10.1177_1947601909358722.pdf]

Supplementary Figure 1

Egfr kinase domain is not mutated in PNET tumors from RT2 mice.

Alignment of the Egfr kinase domain (aa 718-964 / corresponding to exons 18-24), obtained from the mouse genome project (line 1) with the Egfr kinase domain amplified either from C57BL/6J liver or from six Rip-Tag2 tumors. A silent polymorphism (GAA → GAG = Glu → Glu) at position 658 was found in the RT2 background (both in liver cDNA and cDNAs from the 6 independent tumors).

Supplementary Figure 2

Tgf-α mutant mice do not display defects in endocrine pancreatic development

(A-B)) Representative morphology following H&E staining of islets of Langerhans from 10 week-old wild type C57BL/6J mouse (A) vs. a 10 week-old *Tgf-α* $^{wa1/wa1}$ mutant mouse in the same genetic background (B). (C-D) Representative Immunohistochemical staining with an anti-insulin antibody of a pancreatic section from a 10 week-old wild type C57BL/6J mouse (C) vs. a 10 week-old *Tgf-α* $^{wa1/wa1}$ mutant mouse in the same genetic background (D). (E) Glucose tolerance test: average blood glucose levels assayed at defined time points following glucose injection into wild type C57BL/6J mice (Blue line), *Tgf-α* ^{wa1} heterozygous mice (Red line) or *Tgf-α* $^{wa1/wa1}$ mutant mice (Green line). (A-D) Micrographs are representative of multiple fields of sections from three mice of each genotype.

Supplementary Figure 3

Hb-egf mutant mice do not display defects in endocrine pancreatic development

(A-B) Representative morphology following H&E staining of islets of Langerhans from a 12 week-old wild type C3HeB/FeJ mouse (A) vs. a 12 week-old *Hb-egf* mutant mouse in the same genetic background (B). (C-D) Representative Immunohistochemical staining with an anti-insulin antibody of a pancreatic section from a 12 week-old wild type C3HeB/FeJ mouse (C) vs. a 12 week-old *Hb-egf* mutant mouse (D). (E) Glucose tolerance test: average blood glucose levels assayed at defined time points following glucose injection into wild type C3HeB/FeJ mice (Green line), or *Hb-egf* mutant mice (Red line). (A-D) Micrographs are representative of several fields of sections from three mice of each genotype.

Supplementary Figure 4

PNET cancer cells from RT2 mice express phospho-Egfr in vivo and Egfr, Tgf-α and Hb-egf in vitro

(A) Staining of a PNET tumor lesion with secondary antibody (left panel - negative control), or with an anti-pEGFR^{Tyr1068} antibody (right panel); the long exposure was necessary to reveal the positive signal detected in most cancer cells relative to pericytes (> 90% of tumor mass). (B-C) Relative expression of *ErbB-* receptor and *EGF*-ligand family members in cDNAs prepared from total RNA extracts of PNET tumors (blue bars), BTC3 cells (red bars) or BTC4 cells (yellow bars). Levels of mRNAs are expressed as a percentage of the *mGus* control mRNA.

Nolan-Stevaux_Suppl Fig 1

```
1                                                                    60
 Egfr  AAAGTTCTGGGTTCGGGAGCATTTGGCACAGTGTATAAGGGTCTCTGGATCCCAGAAGGT
Liver  AAAGTTCTGGGTTCGGGAGCATTTGGCACAGTGTATAAGGGTCTCTGGATCCCAGAAGGT
RT2.1  AAAGTTCTGGGTTCGGGAGCATTTGGCACAGTGTATAAGGGTCTCTGGATCCCAGAAGGT
RT2.2  AAAGTTCTGGGTTCGGGAGCATTTGGCACAGTGTATAAGGGTCTCTGGATCCCAGAAGGT
RT2.3  AAAGTTCTGGGTTCGGGAGCATTTGGCACAGTGTATAAGGGTCTCTGGATCCCAGAAGGT
RT2.4  AAAGTTCTGGGTTCGGGAGCATTTGGCACAGTGTATAAGGGTCTCTGGATCCCAGAAGGT
RT2.5  AAAGTTCTGGGTTCGGGAGCATTTGGCACAGTGTATAAGGGTCTCTGGATCCCAGAAGGT
RT2.6  AAAGTTCTGGGTTCGGGAGCATTTGGCACAGTGTATAAGGGTCTCTGGATCCCAGAAGGT

       61                                                         120
 Egfr  GAGAAAGTAAAAATCCCGGTGGCCATCAAGGAGTTAAGAGAAGCCACATCTCCAAAAGCC
Liver  GAGAAAGTAAAAATCCCGGTGGCCATCAAGGAGTTAAGAGAAGCCACATCTCCAAAAGCC
RT2.1  GAGAAAGTAAAAATCCCGGTGGCCATCAAGGAGTTAAGAGAAGCCACATCTCCAAAAGCC
RT2.2  GAGAAAGTAAAAATCCCGGTGGCCATCAAGGAGTTAAGAGAAGCCACATCTCCAAAAGCC
RT2.3  GAGAAAGTAAAAATCCCGGTGGCCATCAAGGAGTTAAGAGAAGCCACATCTCCAAAAGCC
RT2.4  GAGAAAGTAAAAATCCCGGTGGCCATCAAGGAGTTAAGAGAAGCCACATCTCCAAAAGCC
RT2.5  GAGAAAGTAAAAATCCCGGTGGCCATCAAGGAGTTAAGAGAAGCCACATCTCCAAAAGCC
RT2.6  GAGAAAGTAAAAATCCCGGTGGCCATCAAGGAGTTAAGAGAAGCCACATCTCCAAAAGCC

       121                                                        180
 Egfr  AACAAAGAAATCCTTGACGAAGCCTATGTGATGGCTAGTGTGGACAACCCTCATGTATGC
Liver  AACAAAGAAATCCTTGACGAAGCCTATGTGATGGCTAGTGTGGACAACCCTCATGTATGC
RT2.1  AACAAAGAAATCCTTGACGAAGCCTATGTGATGGCTAGTGTGGACAACCCTCATGTATGC
RT2.2  AACAAAGAAATCCTTGACGAAGCCTATGTGATGGCTAGTGTGGACAACCCTCATGTATGC
RT2.3  AACAAAGAAATCCTTGACGAAGCCTATGTGATGGCTAGTGTGGACAACCCTCATGTATGC
RT2.4  AACAAAGAAATCCTTGACGAAGCCTATGTGATGGCTAGTGTGGACAACCCTCATGTATGC
RT2.5  AACAAAGAAATCCTTGACGAAGCCTATGTGATGGCTAGTGTGGACAACCCTCATGTATGC
RT2.6  AACAAAGAAATCCTTGACGAAGCCTATGTGATGGCTAGTGTGGACAACCCTCATGTATGC

       181                                                        240
 Egfr  CGCCTCCTGGGCATCTGTCTGACCTCCACTGTCCAGCTCATTACACAGCTCATGCCCTAC
Liver  CGCCTCCTGGGCATCTGTCTGACCTCCACTGTCCAGCTCATTACACAGCTCATGCCCTAC
RT2.1  CGCCTCCTGGGCATCTGTCTGACCTCCACTGTCCAGCTCATTACACAGCTCATGCCCTAC
RT2.2  CGCCTCCTGGGCATCTGTCTGACCTCCACTGTCCAGCTCATTACACAGCTCATGCCCTAC
RT2.3  CGCCTCCTGGGCATCTGTCTGACCTCCACTGTCCAGCTCATTACACAGCTCATGCCCTAC
RT2.4  CGCCTCCTGGGCATCTGTCTGACCTCCACTGTCCAGCTCATTACACAGCTCATGCCCTAC
RT2.5  CGCCTCCTGGGCATCTGTCTGACCTCCACTGTCCAGCTCATTACACAGCTCATGCCCTAC
RT2.6  CGCCTCCTGGGCATCTGTCTGACCTCCACTGTCCAGCTCATTACACAGCTCATGCCCTAC

       241                                                        300
 Egfr  GGTTGCCTCCTGGACTACGTCCGAGAACACAAGGACAACATTGGCTCCCAGTACCTCCTC
Liver  GGTTGCCTCCTGGACTACGTCCGAGAACACAAGGACAACATTGGCTCCCAGTACCTCCTC
RT2.1  GGTTGCCTCCTGGACTACGTCCGAGAACACAAGGACAACATTGGCTCCCAGTACCTCCTC
RT2.2  GGTTGCCTCCTGGACTACGTCCGAGAACACAAGGACAACATTGGCTCCCAGTACCTCCTC
RT2.3  GGTTGCCTCCTGGACTACGTCCGAGAACACAAGGACAACATTGGCTCCCAGTACCTCCTC
RT2.4  GGTTGCCTCCTGGACTACGTCCGAGAACACAAGGACAACATTGGCTCCCAGTACCTCCTC
RT2.5  GGTTGCCTCCTGGACTACGTCCGAGAACACAAGGACAACATTGGCTCCCAGTACCTCCTC
RT2.6  GGTTGCCTCCTGGACTACGTCCGAGAACACAAGGACAACATTGGCTCCCAGTACCTCCTC

       301                                                        360
 Egfr  AACTGGTGTGTGCAGATTGCAAAGGGCATGAACTACCTGGAAGATCGGCGTTTGGTGCAC
Liver  AACTGGTGTGTGCAGATTGCAAAGGGCATGAACTACCTGGAAGATCGGCGTTTGGTGCAC
RT2.1  AACTGGTGTGTGCAGATTGCAAAGGGCATGAACTACCTGGAAGATCGGCGTTTGGTGCAC
RT2.2  AACTGGTGTGTGCAGATTGCAAAGGGCATGAACTACCTGGAAGATCGGCGTTTGGTGCAC
```

```
RT2.3  AACTGGTGTGTGCAGATTGCAAAGGGCATGAACTACCTGGAAGATCGGCGTTTGGTGCAC
RT2.4  AACTGGTGTGTGCAGATTGCAAAGGGCATGAACTACCTGGAAGATCGGCGTTTGGTGCAC
RT2.5  AACTGGTGTGTGCAGATTGCAAAGGGCATGAACTACCTGGAAGATCGGCGTTTGGTGCAC
RT2.6  AACTGGTGTGTGCAGATTGCAAAGGGCATGAACTACCTGGAAGATCGGCGTTTGGTGCAC

       361                                                      420
 Egfr  CGTGACTTGGCAGCCAGGAATGTACTGGTGAAGACACCACAGCATGTCAAGATCACAGAT
Liver  CGTGACTTGGCAGCCAGGAATGTACTGGTGAAGACACCACAGCATGTCAAGATCACAGAT
RT2.1  CGTGACTTGGCAGCCAGGAATGTACTGGTGAAGACACCACAGCATGTCAAGATCACAGAT
RT2.2  CGTGACTTGGCAGCCAGGAATGTACTGGTGAAGACACCACAGCATGTCAAGATCACAGAT
RT2.3  CGTGACTTGGCAGCCAGGAATGTACTGGTGAAGACACCACAGCATGTCAAGATCACAGAT
RT2.4  CGTGACTTGGCAGCCAGGAATGTACTGGTGAAGACACCACAGCATGTCAAGATCACAGAT
RT2.5  CGTGACTTGGCAGCCAGGAATGTACTGGTGAAGACACCACAGCATGTCAAGATCACAGAT
RT2.6  CGTGACTTGGCAGCCAGGAATGTACTGGTGAAGACACCACAGCATGTCAAGATCACAGAT

       421                                                      480
 Egfr  TTTGGGCTGGCCAAACTGCTTGGTGCTGAAGAGAAAGAATATCATGCCGAGGGGGGCAAA
Liver  TTTGGGCTGGCCAAACTGCTTGGTGCTGAAGAGAAAGAATATCATGCCGAGGGGGGCAAA
RT2.1  TTTGGGCTGGCCAAACTGCTTGGTGCTGAAGAGAAAGAATATCATGCCGAGGGGGGCAAA
RT2.2  TTTGGGCTGGCCAAACTGCTTGGTGCTGAAGAGAAAGAATATCATGCCGAGGGGGGCAAA
RT2.3  TTTGGGCTGGCCAAACTGCTTGGTGCTGAAGAGAAAGAATATCATGCCGAGGGGGGCAAA
RT2.4  TTTGGGCTGGCCAAACTGCTTGGTGCTGAAGAGAAAGAATATCATGCCGAGGGGGGCAAA
RT2.5  TTTGGGCTGGCCAAACTGCTTGGTGCTGAAGAGAAAGAATATCATGCCGAGGGGGGCAAA
RT2.6  TTTGGGCTGGCCAAACTGCTTGGTGCTGAAGAGAAAGAATATCATGCCGAGGGGGGCAAA

       481                                                      540
 Egfr  GTGCCTATCAAGTGGATGGCTTTGGAATCAATTTTACACCGAATTTATACACACCAAAGT
Liver  GTGCCTATCAAGTGGATGGCTTTGGAATCAATTTTACACCGAATTTATACACACCAAAGT
RT2.1  GTGCCTATCAAGTGGATGGCTTTGGAATCAATTTTACACCGAATTTATACACACCAAAGT
RT2.2  GTGCCTATCAAGTGGATGGCTTTGGAATCAATTTTACACCGAATTTATACACACCAAAGT
RT2.3  GTGCCTATCAAGTGGATGGCTTTGGAATCAATTTTACACCGAATTTATACACACCAAAGT
RT2.4  GTGCCTATCAAGTGGATGGCTTTGGAATCAATTTTACACCGAATTTATACACACCAAAGT
RT2.5  GTGCCTATCAAGTGGATGGCTTTGGAATCAATTTTACACCGAATTTATACACACCAAAGT
RT2.6  GTGCCTATCAAGTGGATGGCTTTGGAATCAATTTTACACCGAATTTATACACACCAAAGT

       541                                                      600
 Egfr  GATGTCTGGAGCTATGGTGTCACTGTGTGGGAACTGATGACCTTTGGGTCCAAGCCTTAT
Liver  GATGTCTGGAGCTATGGTGTCACTGTGTGGGAACTGATGACCTTTGGGTCCAAGCCTTAT
RT2.1  GATGTCTGGAGCTATGGTGTCACTGTGTGGGAACTGATGACCTTTGGGTCCAAGCCTTAT
RT2.2  GATGTCTGGAGCTATGGTGTCACTGTGTGGGAACTGATGACCTTTGGGTCCAAGCCTTAT
RT2.3  GATGTCTGGAGCTATGGTGTCACTGTGTGGGAACTGATGACCTTTGGGTCCAAGCCTTAT
RT2.4  GATGTCTGGAGCTATGGTGTCACTGTGTGGGAACTGATGACCTTTGGGTCCAAGCCTTAT
RT2.5  GATGTCTGGAGCTATGGTGTCACTGTGTGGGAACTGATGACCTTTGGGTCCAAGCCTTAT
RT2.6  GATGTCTGGAGCTATGGTGTCACTGTGTGGGAACTGATGACCTTTGGGTCCAAGCCTTAT

       601                                                      660
 Egfr  GATGGAATCCCAGCAAGTGACATCTCATCCATCCTAGAGAAAGGAGAACGCCTTCCACAG
Liver  GATGGAATCCCAGCAAGTGACATCTCATCCATCCTAGAGAAAGGAGAGCGCCTTCCACAG
RT2.1  GATGGAATCCCAGCAAGTGACATCTCATCCATCCTAGAGAAAGGAGAGCGCCTTCCACAG
RT2.2  GATGGAATCCCAGCAAGTGACATCTCATCCATCCTAGAGAAAGGAGAGCGCCTTCCACAG
RT2.3  GATGGAATCCCAGCAAGTGACATCTCATCCATCCTAGAGAAAGGAGAGCGCCTTCCACAG
RT2.4  GATGGAATCCCAGCAAGTGACATCTCATCCATCCTAGAGAAAGGAGAGCGCCTTCCACAG
RT2.5  GATGGAATCCCAGCAAGTGACATCTCATCCATCCTAGAGAAAGGAGAGCGCCTTCCACAG
RT2.6  GATGGAATCCCAGCAAGTGACATCTCATCCATCCTAGAGAAAGGAGAGCGCCTTCCACAG

       661                                                      720
 Egfr  CCACCTATCTGCACCATCGATGTCTACATGATCATGGTCAAGTGCTGGATGATAGATGCT
Liver  CCACCTATCTGCACCATCGATGTCTACATGATCATGGTCAAGTGCTGGATGATAGATGCT
```

```
RT2.1   CCACCTATCTGCACCATCGATGTCTACATGATCATGGTCAAGTGCTGGATGATAGATGCT
RT2.2   CCACCTATCTGCACCATCGATGTCTACATGATCATGGTCAAGTGCTGGATGATAGATGCT
RT2.3   CCACCTATCTGCACCATCGATGTCTACATGATCATGGTCAAGTGCTGGATGATAGATGCT
RT2.4   CCACCTATCTGCACCATCGATGTCTACATGATCATGGTCAAGTGCTGGATGATAGATGCT
RT2.5   CCACCTATCTGCACCATCGATGTCTACATGATCATGGTCAAGTGCTGGATGATAGATGCT
RT2.6   CCACCTATCTGCACCATCGATGTCTACATGATCATGGTCAAGTGCTGGATGATAGATGCT

        721                       742
 Egfr   GATAGCCGCCCAAAGTTCCGAG
Liver   GATAGCCGCCCAAAGTTCCGAG
RT2.1   GATAGCCGCCCAAAGTTCCGAG
RT2.2   GATAGCCGCCCAAAGTTCCGAG
RT2.3   GATAGCCGCCCAAAGTTCCGAG
RT2.4   GATAGCCGCCCAAAGTTCCGAG
RT2.5   GATAGCCGCCCAAAGTTCCGAG
RT2.6   GATAGCCGCCCAAAGTTCCGAG
```

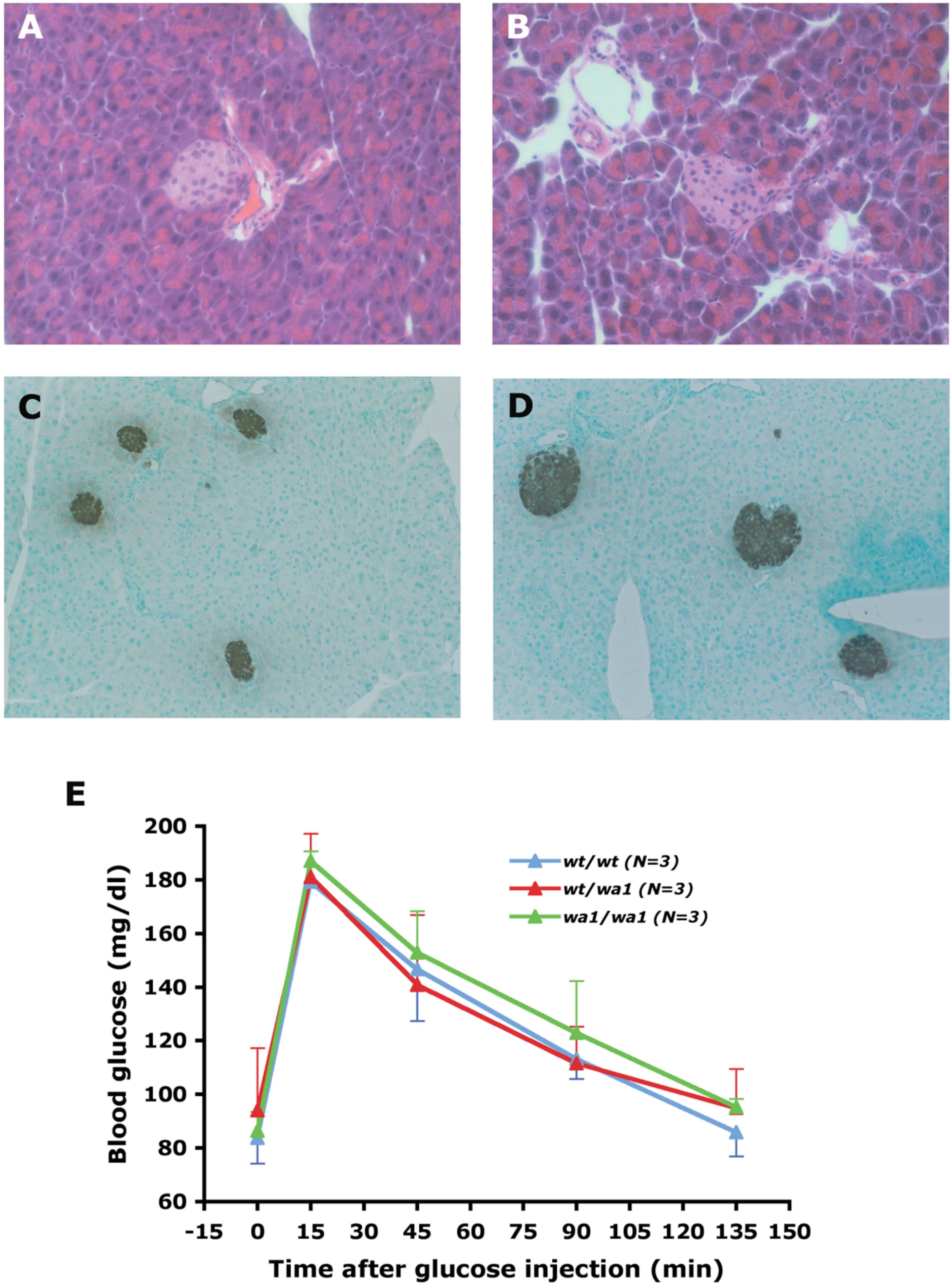
A
B
C
D
E
Blood glucose (mg/dl)
200
180
160
140
120
100
80
60
-15
0
15
30
45
60
75
90
105
120
135
150
Time after glucose injection (min)
wt/wt (N=3)
wt/wa1 (N=3)
wa1/wa1 (N=3)

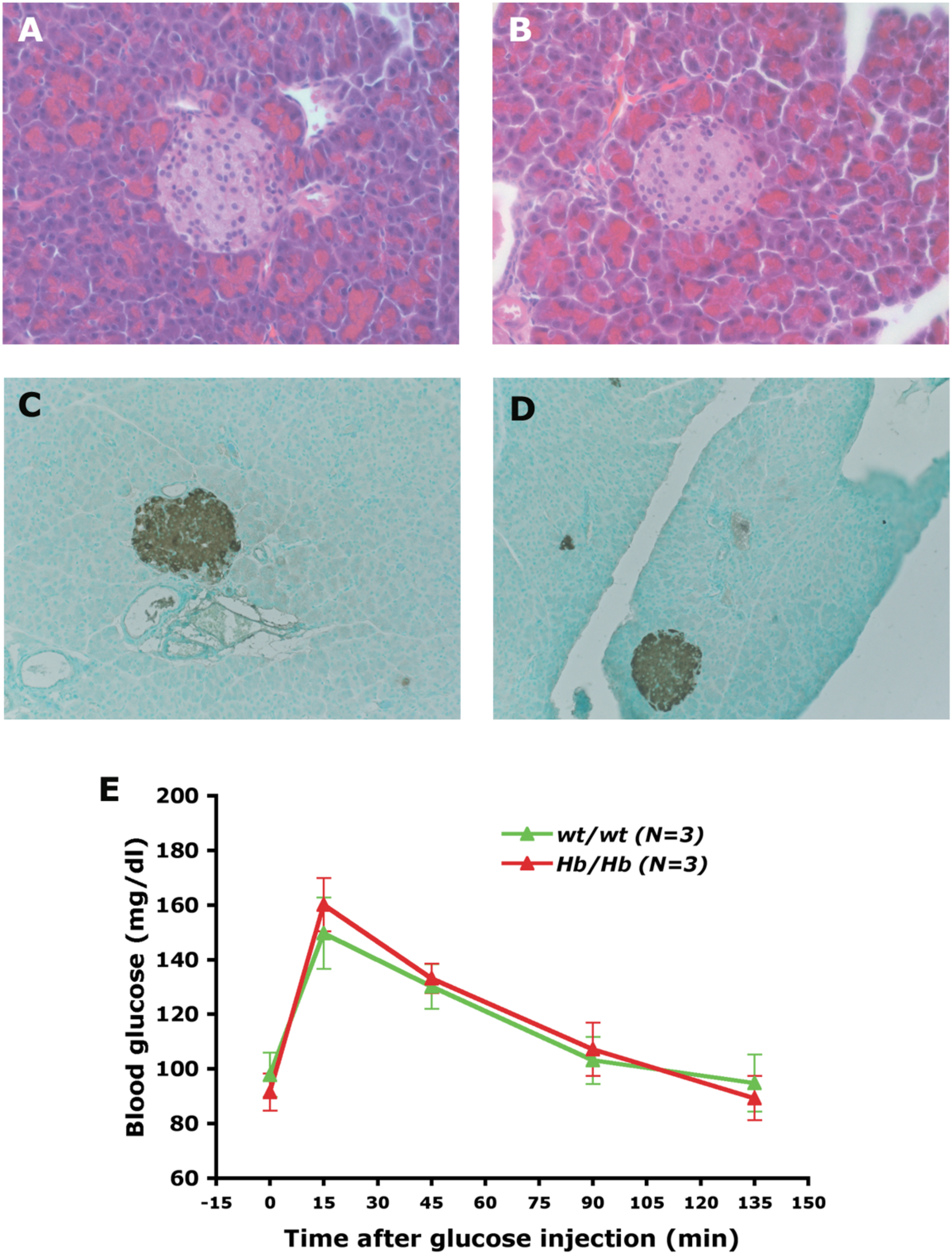

A
B
C
D
E
Blood glucose (mg/dl)
200
180
160
140
120
100
80
60
-15
0
15
30
45
60
75
90
105
120
135
150
Time after glucose injection (min)
wt/wt (N=3)
Hb/Hb (N=3)

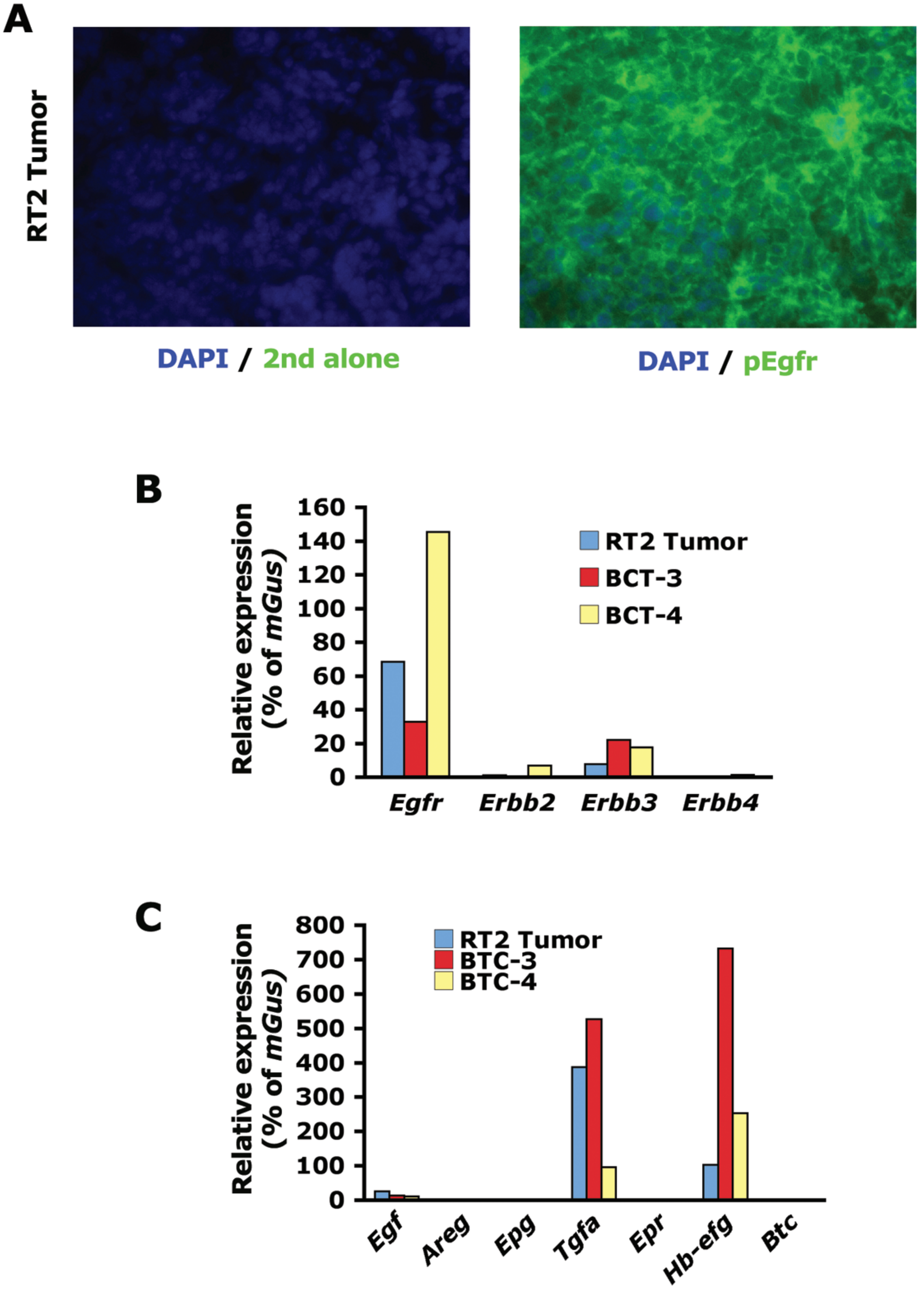
A
RT2 Tumor
DAPI / 2nd alone
DAPI / pEgfr
B
Relative expression (% of mGus)
0
20
40
60
80
100
120
140
160
RT2 Tumor
BCT-3
BCT-4
Egfr
Erbb2
Erbb3
Erbb4
C
Relative expression (% of mGus)
0
100
200
300
400
500
600
700
800
RT2 Tumor
BTC-3
BTC-4
Egf
Areg
Epg
Tgfa
Epr
Hb-efg
Btc
